# Effector memory CD8 T-cells as a novel peripheral blood biomarker for activated T-cell pediatric acute liver failure

**DOI:** 10.1371/journal.pone.0286394

**Published:** 2023-06-02

**Authors:** Catherine A. Chapin, Thomas M. Burn, Tamir Diamond, Kathleen M. Loomes, Estella M. Alonso, Edward M. Behrens

**Affiliations:** 1 Department of Pediatrics, Feinberg School of Medicine, Ann & Robert H. Lurie Children’s Hospital of Chicago, Northwestern University, Chicago, Illinois, United States of America; 2 Department of Pediatrics, Perelman School of Medicine, The Children’s Hospital of Philadelphia, University of Pennsylvania, Philadelphia, Pennsylvania, United States of America; Massachusetts General Hospital, Harvard Medical School, UNITED STATES

## Abstract

A distinct phenotype of pediatric acute liver failure (PALF) has been identified, labeled activated T-cell hepatitis. These patients, previously included within the indeterminate group, have evidence of systemic immune activation and liver biopsy specimens with dense infiltration of CD8+ T-cells. We aimed to evaluate the peripheral blood T-cell phenotype in PALF patients with activated T-cell hepatitis compared to indeterminate cause. PALF patients with unknown etiology age 1–17 years were prospectively enrolled between 2017–2020. Within the unknown group, patients were classified as either activated T-cell hepatitis if they had a liver biopsy with dense or moderate CD8 staining and an elevated soluble interleukin-2 receptor level, or they were classified as indeterminate if they did not meet these criteria. Whole blood was collected for flow cytometry and T-cell phenotyping. Four patients with activated T-cell hepatitis and 4 patients with indeterminate PALF were enrolled. Activated T-cell hepatitis patients had significantly greater percentage of CD8 T-cells that were effector memory (T_EM_) phenotype compared to indeterminate PALF patients (median 66.8% (IQR 57.4–68.7) vs 19.1% (IQR 13.4–25.2), *P* = 0.03). In addition, CD8+ T_EM_ cells in activated T-cell hepatitis patients were significantly more likely to be CD103 positive, a marker of tissue resident memory T-cells, compared to indeterminate PALF patients (median 12.4% (IQR 9.5–14.7) vs 4.7% (IQR 4.5–5.3), *P* = 0.03). We found patients with activated T-cell hepatitis can be identified by the unique pattern of increased percentage of peripheral blood effector memory CD8+ CD103+ T-cells. These findings will guide future studies exploring the T-cell phenotype for these patients and whether they may respond to directed immunosuppressive therapies.

## Introduction

Many children with indeterminate pediatric acute liver failure (PALF) have evidence of immune-mediated liver injury driven by untethered inflammatory responses [1, 2]. Patients often share a phenotype of immune activation including overproduction of inflammatory cytokines, high serum soluble interleukin-2 receptor (sIL-2R) levels, peripheral blood cytopenias, and an increased risk of developing acquired aplastic anemia [2–5]. In prior studies in both a discovery and validation cohort, we found that dense hepatic CD8 T-cell, CD103 (a marker of tissue resident memory T-cells), and perforin staining is a distinctive histopathologic feature of liver tissue specimens from children with indeterminate PALF [6, 7]. We proposed that patients with a prominent CD8+ infiltrate on liver biopsy and no alternative etiology identified be classified as activated T-cell hepatitis with or without acute liver failure (ALF) [7]. However, while liver biopsy can be safely performed in PALF patients [8], this procedure is not always performed and tissue samples may not be available for CD8 staining. There remains a critical need for more widely available blood-based biomarkers to identify these patients, who may respond to treatment with immunosuppressive therapies [4, 9]. In this study, we aimed to describe the peripheral blood T-cell phenotype of PALF patients of unknown etiology. In addition, we sought to determine whether the distinctive T-cell phenotype seen in liver tissue from activated T-cell hepatitis patients was also present in peripheral blood.

## Materials and methods

This was a prospective study of children with PALF of unknown etiology age 1 through 17 years admitted to Ann & Robert H. Lurie Children’s Hospital of Chicago (LCH) between August 2017 and February 2020. PALF was defined using established Pediatric Acute Liver Failure Study Group (PALFSG) criteria: 1) no known evidence of chronic liver disease; 2) biochemical evidence of severe liver injury occurring within 8 weeks of illness onset; and 3) hepatic-based coagulopathy (not corrected by vitamin K) defined as prothrombin time (PT) ≥ 15 seconds or international normalized ratio (INR) ≥ 1.5 in the presence of hepatic encephalopathy (HE) or a PT ≥ 20 seconds or INR ≥ 2.0 with or without HE [10].

Patients were considered eligible for enrollment if a known etiology for their liver failure was not identified within the first 48–72 hours of admission. Patients were classified as indeterminate PALF if no etiology was identified by time of hospital discharge despite an age-appropriate diagnostic evaluation. Previously included within the indeterminate PALF category, we now consider activated T-cell hepatitis as a separate group. Patients were classified as activated T-cell hepatitis if they met our center criteria for this diagnosis which includes a liver biopsy with predominant portal and lobular CD8+ T-cell inflammation, sIL2R level elevated above the upper limit of normal, and no other cause of their ALF identified. All patients received a standard age-appropriate diagnostic evaluation which included testing for acute viral hepatitis (Hepatitis A, Hepatitis B, Hepatitis C, Epstein-Barr virus, and Cytomegalovirus), serum acetaminophen level and medication and supplement history, screening for autoimmune hepatitis (AIH) (anti-nuclear antibody, anti-smooth muscle antibody, and anti-liver kidney microsomal antibody), screening for Wilson disease (ceruloplasmin level) and screening for metabolic disorders (urine organic acids and acylcarnitine profile). Immune marker labs were obtained to evaluate for hemophagocytic lymphohistiocytosis (HLH) and overall immune activation (sIL-2R level, perforin and granzyme B assay, natural killer cell function, and T- and B-cell flow cytometry). Additional diagnostic studies were performed as indicated based on the clinical scenario.

Patient demographic, clinical, laboratory, and outcome data were collected from the electronic medical record and included age at presentation, gender, 21-day outcome from admission, and development of aplastic anemia within one year prior to or following PALF presentation. Clinical labs were collected as available. If liver biopsy was done as part of clinical care, immunohistochemical (IHC) staining was performed by the LCH clinical pathology laboratory. Formalin-fixed paraffin-embedded liver tissue specimens were stained with antibody for CD8 T-cells (CD8) and intensity of immunohistochemical staining was subjectively scored by investigators as minimal, moderate, or dense as previously described [6] with staining score agreed upon by the same two investigators including one pediatric liver pathologist. During this study, patients classified as activated T-cell hepatitis were considered for treatment with intravenous corticosteroids at the discretion of the clinical care team [7].

Patients enrolled in this study had additional research whole blood collected which was sent to CHOP for flow cytometry and T-cell phenotyping. Whole blood samples were collected within 48 hours of study enrollment and shipped ambient overnight to the Behrens laboratory at CHOP for lymphocyte isolation and flow cytometry studies. For patients who received corticosteroids, samples were collected prior to initiation of treatment. Isolated lymphocytes were stained with LIVE/DEAD fixable viability dye from Life Technologies (now ThermoScientific, Waltham, MA), Fc block (BD Biosciences, San Diego, CA), and CD3, HLA-DR, CD19, CD14, CD56, CD4, CD8, CD103, CD45, CD45RA, and chemokine (C-C motif) receptor 7 (CCR7) antibodies (BD Biosciences, San Diego, CA). Following surface antigen staining, cells were stained for perforin (BD Biosciences) using the Cytofix/Cytoperm kit (BD Biosciences).

Statistical analyses were conducted using GraphPad Prism version 9.3 for Windows GraphPad Software (La Jolla California USA, www.graphpad.com). Data are reported as percentages if categorical or medians with interquartile range if not normally distributed. Wilcoxon rank-sum and Fisher’s exact tests were used to compare patient characteristics, flow cytometry, and immune studies results between groups. Samples were acquired on a LSRFortessa (BD Biosciences) or MACSQuant Analyzer 10 (Miltenyi Biotec) flow cytometer and analyzed using FlowJo software version 10.6 (Tree Star). P-values < 0.05 were considered statistically significant.

The study was approved by the Ann & Robert H. Lurie Children’s Hospital of Chicago (NO: 2017–820) and Children’s Hospital of Philadelphia (NO: 15–011950) Institutional Review Boards. Written informed consent was obtained for all patients and written assent was obtained for all patients 12 years of age and older.

## Results

During the time-period of this study, 29 children presented to LCH who met PALF criteria. Thirteen were excluded due to age. Six were excluded due to diagnoses of intentional acetaminophen overdose (n = 5) and autoimmune hepatitis (n = 1) identified during the first 72 hours of admission. The remaining 10 patients did not have an etiology for their liver failure identified and were enrolled in our study. Two patients had a diagnosis made after enrollment and were excluded from primary analyses: 1 was diagnosed with acute acetaminophen toxicity (based on patient admitting to intentional overdose after recovery and liver biopsy with characteristic centrilobular necrosis) and 1 was diagnosed with acute hepatitis E infection (based on positive Hepatitis E IgM and history of recent travel to India). Four patients met criteria for activated T-cell hepatitis. The other 4 patients did not have an etiology identified and remained indeterminate PALF at time of hospital discharge and at last outpatient follow up visit.

**Fig 1:**
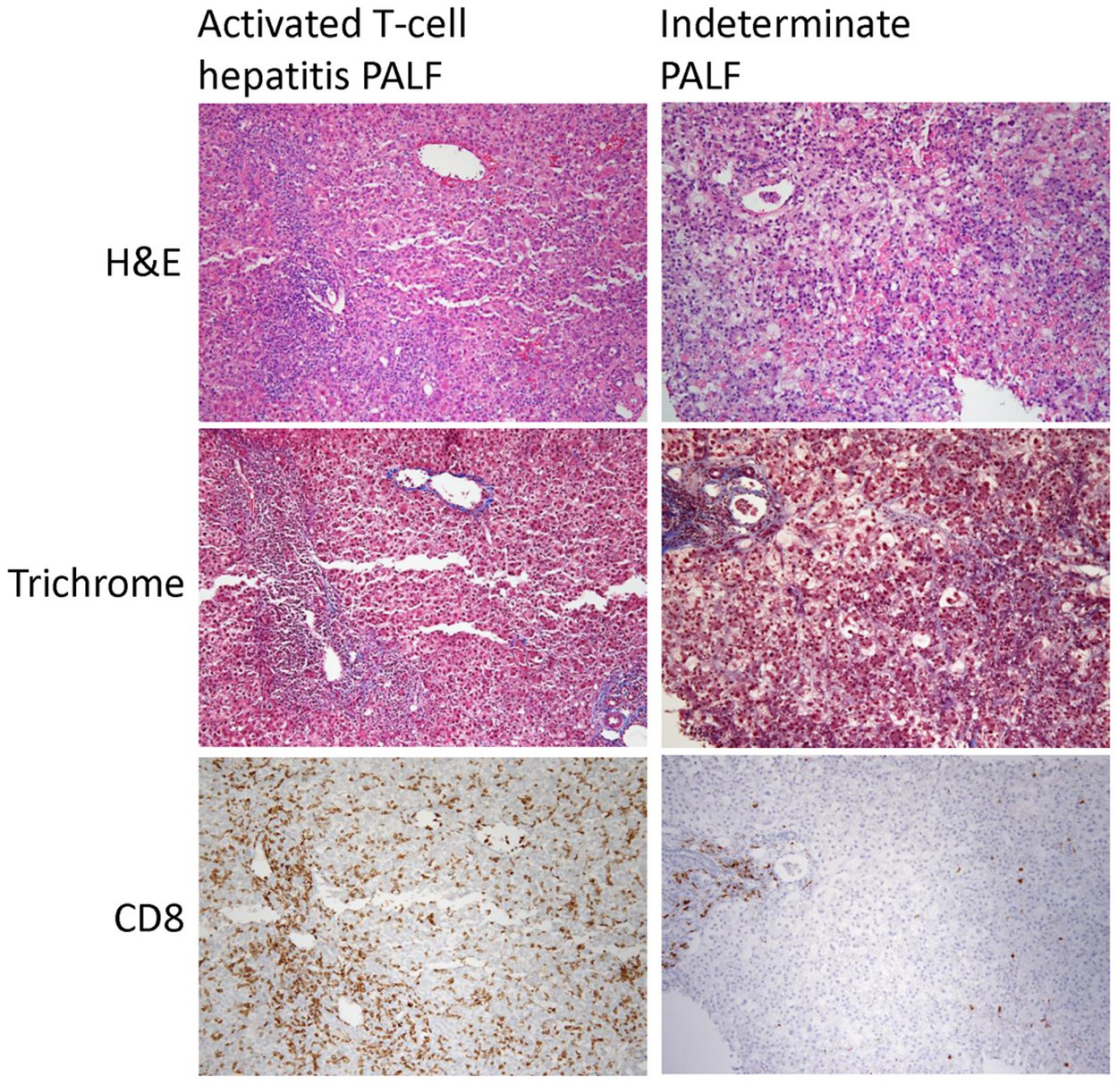
Liver biopsy images at 10x magnification of representative hematoxylin and eosin (H&E), trichrome, and CD8 immunohistochemical staining patterns from an activated T-cell hepatitis PALF case with dense CD8 staining and an indeterminate PALF case with minimal CD8 staining.

Patient demographics, clinical characteristics, and laboratory values are displayed in Table 1. Patients with activated T-cell hepatitis were younger median age 4.0 (IQR 3.0–7.0) vs 10.0 (6.3–12.8) years, but this was not statistically significant. Activated T-cell hepatitis patients had a trend toward longer duration of symptoms prior to hospital admission (3.0 (IQR 2.8–3.0) vs 0.8 (IQR 0.5–1.3) weeks, *P* = 0.06) and significantly higher enrollment total bilirubin levels (18.6 (IQR 17.5–19.8) vs 2.6 (IQR 1.1–6.6) mg/dL, *P* = 0.03) compared to indeterminate PALF patients. Other clinical characteristics were similar between groups. Patients in the indeterminate group received additional supports including ventilator support (n = 2), continuous hemodialysis (n = 1) and vasopressor support (n = 1) while these were not needed for any of the activated T-cell hepatitis patients. Two patients in the activated T-cell hepatitis group developed aplastic anemia, compared to none in the indeterminate PALF group. These two patients both had additional workup done related to their aplastic anemia diagnosis, which was unrevealing, including a negative bone marrow failure syndrome genetic panel. Liver biopsy CD8 T-cell staining was scored as dense (n = 2) or moderate (n = 2) in the activated T-cell hepatitis patients. For the 2 indeterminate PALF patients who had a liver biopsy performed, one had minimal CD8 staining and the other had moderate CD8 staining but did not have an elevated sIL-2R level to meet criteria for the activated T-cell hepatitis group. Example liver biopsy images from a representative activated T-cell hepatitis and indeterminate PALF case are shown in Fig 1.

**Table 1:** Patient demographic, laboratory, and clinical characteristics.

	Total Cases	Activated T-cell hepatitis	Indeterminate PALF	P value
	n = 8	n = 4	n = 4	
***At Study Enrollment***				
Age, years	6.5 (3.0–12.3)	4.0 (3.0–7.0)	10.0 (6.3–12.8)	0.63
Gender, male	4 (50)	2 (50)	2 (50)	>0.99
Symptoms prior to admission, weeks	2.0 (0.9–3.0)	3.0 (2.8–3.0)	0.8 (0.5–1.3)	0.06
Hepatic encephalopathy level 0–1	5 (63)	3 (75)	2 (50)	>0.99
Alanine aminotransferase (IU/L)	2167 (1279–5317)	1576 (1276–1965)	6545 (3303–9800)	0.20
Total bilirubin (mg/dL)	14.6 (3.3–18.5)	18.6 (17.5–19.8)	2.6 (1.1–6.6)	0.03
International normalized ratio	2.1 (2.1–2.3)	2.1 (2.0–2.1)	2.3 (2.1–3.0)	0.48
Total white blood cell count (1000/mm^3^)	4.0 (3.2–8.0)	3.0 (2.2–4.9)	6.1 (4.2–9.6)	0.20
Hemoglobin (g/dL)	11.2 (10.2–12.7)	10.6 (10.2–11.6)	11.9 (10.8–12.8)	0.68
Platelet count (1000/mm^3^)	183 (150–242)	183 (150–218)	199 (131–256)	>0.99
***Worst values during admission***				
Hepatic encephalopathy level 2–4	4 (50)	2 (50)	2 (50)	>0.99
Highest alanine aminotransferase (IU/L)	2381 (1513–7249)	1876 (1513–2249)	7833 (5283–9988)	0.34
Highest total bilirubin (mg/dL)	20.4 (4.1–22.1)	21.6 (20.4–22.6)	3.2 (1.1–9.2)	0.11
Highest international normalized ratio	2.3 (2.2–2.5)	2.3 (2.2–2.4)	2.3 (2.2–3.0)	0.86
Lowest total white blood cell count (1000/mm^3^)	1.7 (1.3–3.7)	1.7 (1.3–5.5)	1.9 (1.3–3.7)	>0.99
Lowest hemoglobin (g/dL)	10.3 (7.5–11.4)	10.3 (9.4–11.0)	9.2 (7.3–11.6)	0.89
Lowest platelet count (1000/mm^3^)	95 (48–179)	140 (100–179)	60 (44–115)	0.68
***Outcome and follow up***				
Duration of admission, days	12.0 (7.8–16.3)	9.0 (7.8–11.3)	13.5 (6.3–18.5)	0.48
Admission to normal liver enzymes, days	92.0 (57.5–107.0)	110.0 (92.0–130.0)	52.0 (39.5–72.3)	0.11
Developed aplastic anemia	2 (25)	2 (50)	0	0.43

^a^Data are medians (interquartile range) or n (%).

**Fig 2:**
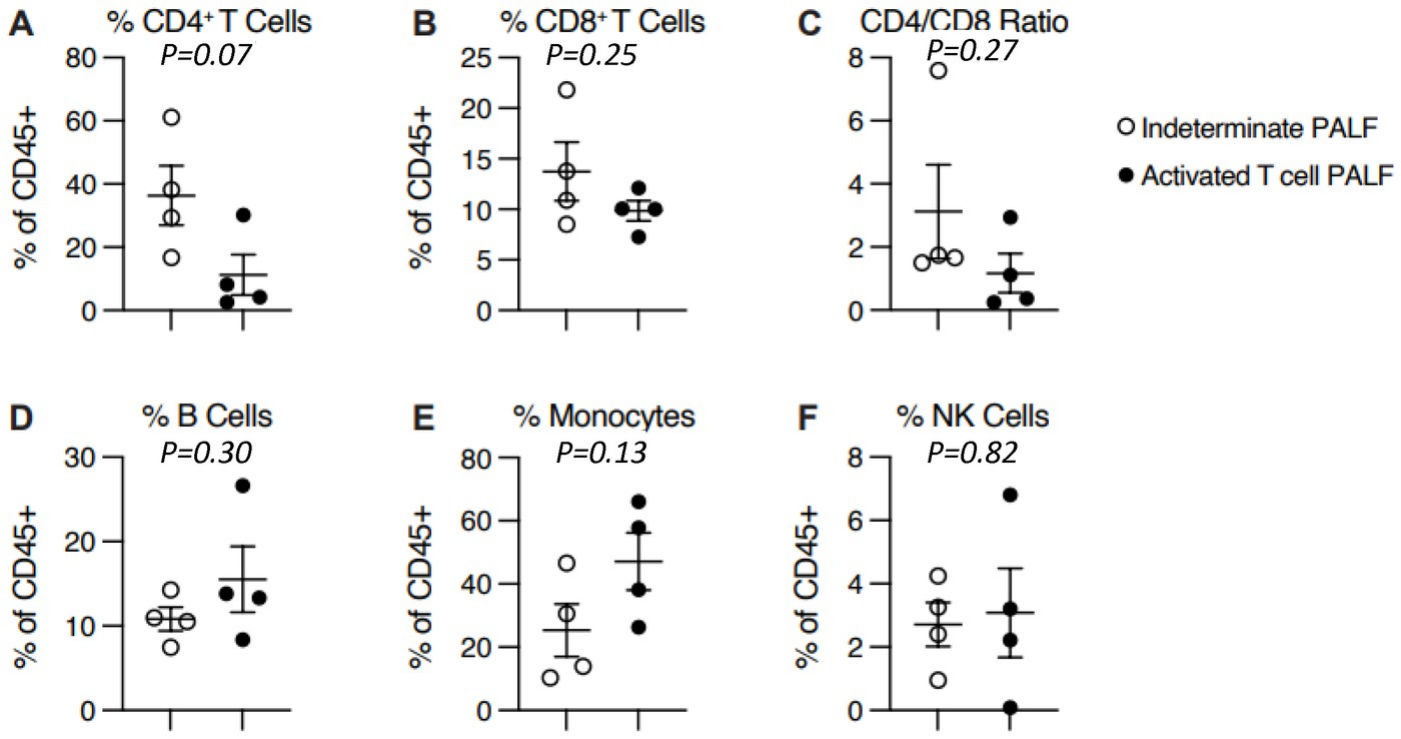
Flow cytometry peripheral blood immune cell subsets do not differ between activated T-cell PALF and indeterminate PALF. No difference between %CD4+ T-cells (A) or %CD8+ T-cells (B) between groups. (C) Non-significant trend toward lower CD4/CD8 T-cell ratio in activated T-cell hepatitis PALF group (*P* = 0.27). No difference between %B cells (D), %Monocytes (E), or %NK cells between groups. Results quantified as mean ± SEM. Symbols denote individual patient samples.

**Fig 3:**
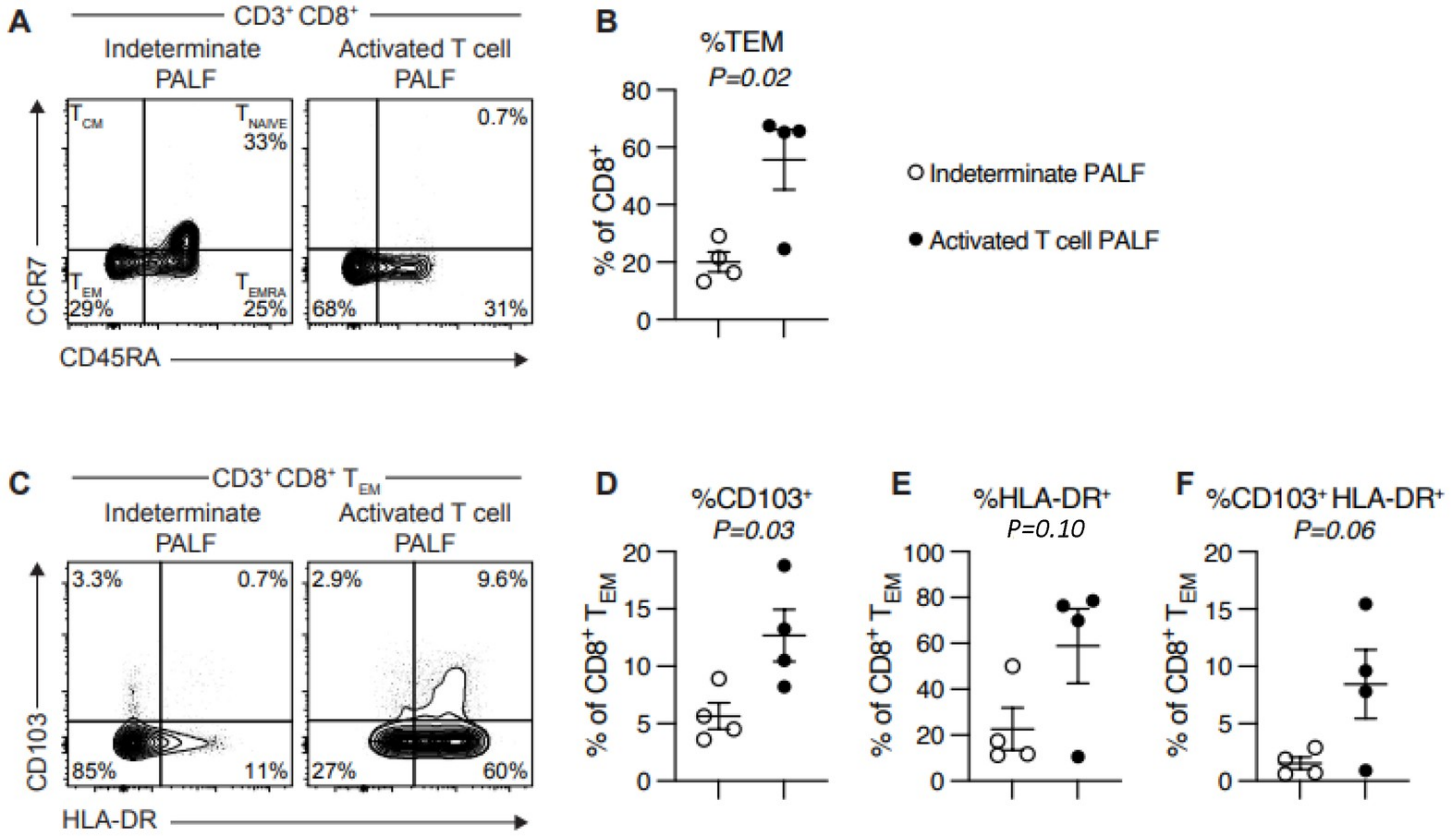
Activated T-cell PALF patients have an increased percentage of peripheral blood CD8+ effector memory T-cells (T_EM_) and CD103+ CD8+ T_EM_ cells. (A & B) Activated T-cell hepatitis PALF patients have significant increase in %CD8+T_EM_ (CD45RA^Lo^ and CCR7^Lo^) (*P* = 0.02). (C & D) Activated T-cell PALF patients have significantly higher %CD8+T_EM_ cells with marker of tissue residency (CD103+) (*P* = 0.03). Patients with activated T-cell PALF showed increase in %HLA-DR+ in total CD8+T_EM_ population (E) and specifically in CD8+T_EM_CD103+ population (F) but did not reach statistical significance. Results quantified as mean ± SEM. Symbols denote individual patient samples.

**Fig 4:**
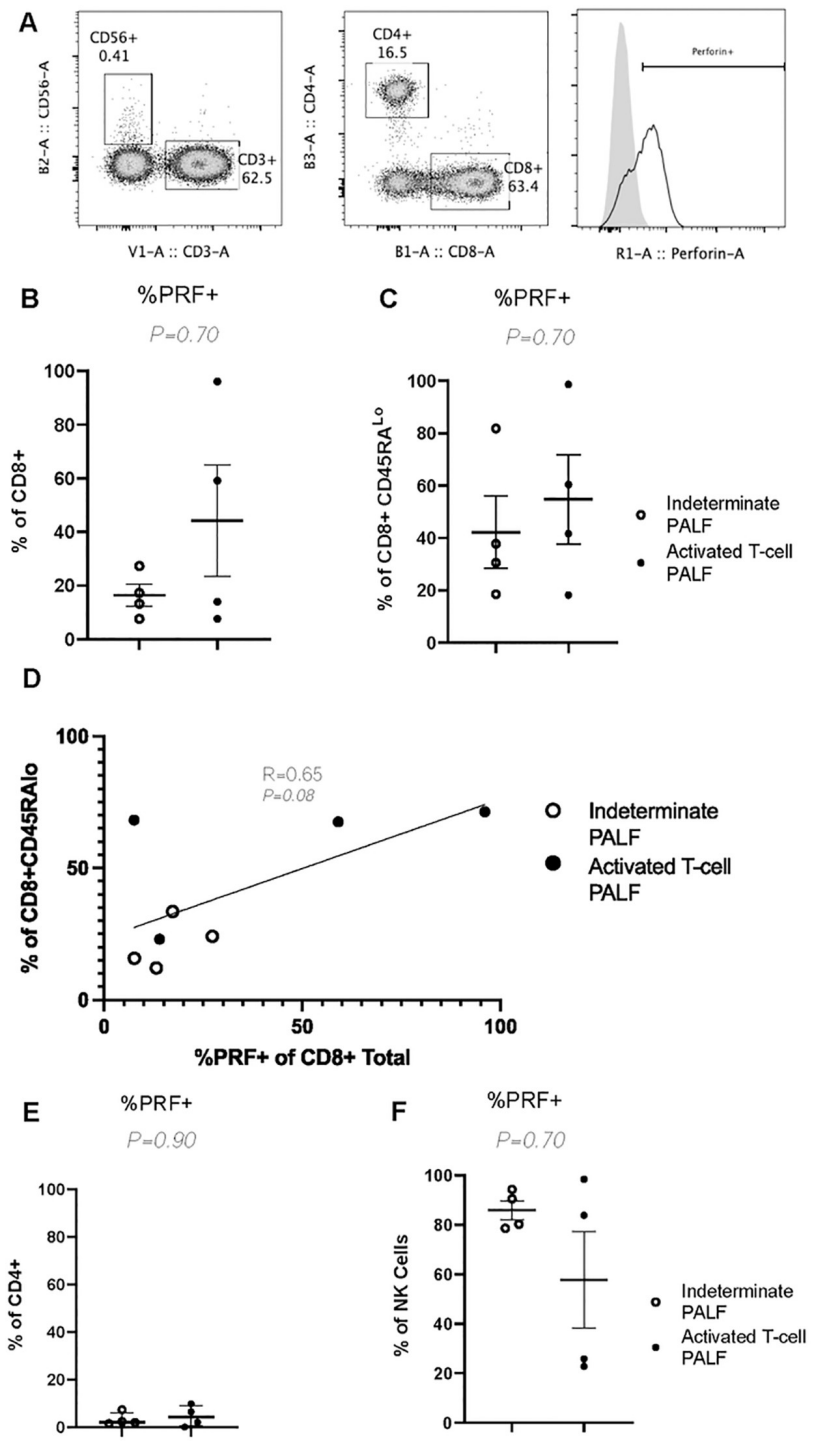
Lymphocyte perforin staining does not help distinguish activated T-cell PALF from indeterminate PALF. (A) Example of gating strategy of percent perforin positive (%PRF+) from CD8+ T-cells (Lymphocytes, Live, CD3+, CD56-, CD8+, CD4-). Grey histogram depicts isotype and clear histogram perforin staining. (B-C) Quantification of %PRF+ cells from CD8+ (B), CD8+ CD45RA^Lo^ (C). (D) Linear regression of %PRF+ CD8+ T cells to total percent of CD8+CD45RA^Lo^ cells with correlation coefficient and significance (*P*-value) embedded in graph. Quantification of %PRF+ cells from CD4+ (E) and NK Cells (F) with results quantified as mean ± SEM. Symbols denote individual patient samples.

Clinical and research peripheral blood immune studies are displayed in Table 2. Clinical T- and B-cell flow cytometry studies were notable for a trend toward lower CD4:CD8 ratio (0.3 (IQR 0.3–0.7) vs 1.8 (IQR 1.5–2.6), *P* = 0.11) and increased percentage of HLA-DR+ (activated) T-cells (32.0% (IQR 23.8–37.3) vs 8.0% (IQR 4.8–11.5), *P* = 0.14) in the activated T-cell hepatitis compared to indeterminate PALF group. The results of research flow cytometry studies for all 8 patients are shown in Figs 2–4. There was no difference in percent CD4+ or CD8+ T-cells, or any other immune cell subsets, between groups (Fig 2). In agreement with clinical flow cytometry, there was a non-significant trend toward lower CD4:CD8 ratio in the activated T-cell hepatitis patients (0.8 (IQR 0.33–1.62)) compared to the indeterminate PALF patients (1.8 (IQR 1.7–3.3), *P* = 0.27) (Fig 2C). Activated T-cell hepatitis patients had a significantly greater percentage of CD8 T-cells that were effector memory (T_EM_) phenotype compared to indeterminate PALF patients (66.8% (IQR 57.4–68.7) vs 19.1% (IQR 13.4–25.2), *P* = 0.03) (Fig 3A and 3B). In addition, the CD8+ T_EM_ cells in activated T-cell hepatitis patients were significantly more likely to be positive for the tissue residency marker CD103, even though they are circulating, compared to indeterminate PALF patients (12.4% (IQR 9.5–14.7) vs 4.7% (IQR 4.5–5.3), *P* = 0.03) (Fig 3C and 3D). There was a non-significant trend towards increased percent of CD8+ T_EM_ cells that were HLA-DR+ (18.7% (IQR 14.1–51.3) vs 77.2% (IQR 35.9–82.2), *P* = 0.06) and increased percent of CD8+ T_EM_ cells that were both HLA-DR+ and CD103+ (1.3% (IQR 0.6–2.7) vs 8.7% (IQR 2.6–14.0), *P* = 0.10) in the activated T-cell hepatitis compared to indeterminate PALF group (Fig 3E and 3F). We also examined perforin expression, a marker of cell cytolytic activity, and found there was no difference in CD8 T-cell perforin expression in activated T-cell hepatitis patients compared to indeterminate PALF (36.6% (IQR 7.6–86.9) vs 15.3% (IQR 7.62–24.8), *P* = 0.70) (Fig 4A–4C). To determine if perforin positivity was simply another marker of memory status, we correlated percent perforin positive of total CD8+ T-cells with percent CD45RA^lo^ (Fig 4D). Among all patients, there was not a statistically significant correlation, and within the activated T-cell hepatitis PALF group, there was no correlation between perforin expression and T-cell memory status suggesting these are measuring different aspects of the physiology. Finally, we noted no differences, as expected, in perforin expression in CD4+ T-cells or NK cells (Fig 4E and 4F). For these analyses we excluded the 2 patients who had diagnoses identified after enrollment in the study, however, if they are included in the indeterminate group the results remain similar (S1 File).

**Table 2:** Clinical and research immune studies laboratory data.

	All Patients	Activated T-cell hepatitis	Indeterminate PALF	P value
	n = 8	n = 4	n = 4	
Soluble IL-2 receptor (IU/mL)	2116 (1689–6433)	4588 (1689–8653)	2116 (1875–3135)	0.70
Perforin and Granzyme B Assay, n	6	4	2	
% CD8 T-cells Perforin+	23.5 (12.5–33.0)	29.5 (18.5–44.0)	16.5 (12.8–20.3)	0.80
% CD8 T-cells Granzyme+	43.5 (21.5–63.3)	60.5 (44.5–70.0)	25.0 (21.5–28.5)	0.53
Clinical T & B cell quantitation				
CD4 to CD8 T-cell ratio	1.4 (0.3–1.8)	0.3 (0.3–0.7)	1.8 (1.5–2.6)	0.11
% CD8+ T-cells	28.0 (23.5–34.8)	34.5 (26.5–40.3)	26.0 (21.3–28.5)	0.34
% HLA-DR+ T-cells	12.0 (5.0–31.0)	32.0 (23.8–37.3)	8.0 (4.8–11.5)	0.14
Research Flow cytometry				
CD4 to CD8 T-cell ratio	1.6 (1.0–2.1)	0.8 (0.3–1.6)	1.8 (1.7–3.3)	0.20
% CD8+ T-cells	34.5 (29.3–42.2)	46.3 (34.7–56.2)	32.4 (26.1–34.1)	0.20
% Effector memory CD8+ T-cells	29.5 (21.6–66.6)	66.8 (57.4–68.7)	19.1 (13.4–25.2)	0.03
% Effector memory CD8+ CD103+ T-cells	7.1 (4.7–11.3)	12.4 (9.5–14.7)	4.7 (4.5–5.3)	0.03

^a^Data are medians (interquartile range).

Within the activated T-cell hepatitis group, 3 of the 4 patients were ≤ 5 years old at time of enrollment and had certain features that the 4^th^ teenage patient did not. Specifically, the 3 younger patients all presented with co-existent cytopenias (enrollment WBC 2.5 1000/mm^3^ (IQR 2.0–3.0) and platelet count 157 1000/mm^3^ (IQR 142–183)) and CD4:CD8 T-cell ratio < 1.0 (0.3 (IQR 0.25–0.31)). Of those 3 patients, 2 developed aplastic anemia (diagnosed by bone marrow biopsy), with one managed with transfusions alone and one requiring stem cell transplantation. In addition, the older patient had a research flow cytometry pattern more similar to the indeterminate PALF group rather than the activated T-cell hepatitis group. This raises the question as to whether the teenage patient may have had a milder phenotype or a different pathophysiology for their liver disease. Clinical and research immune studies data for the 3 younger activated T-cell hepatitis patients compared to the 4 indeterminate PALF patients is presented in S1 File. Comparisons were non-significant, likely related to small patient numbers, however there was a trend toward an increase in effector memory CD8 T-cells that are CD103+ and HLA-DR+ in the activated T-cell hepatitis group.

All patients had recovery with their native liver. The activated T-cell hepatitis patients were all treated with corticosteroids at the discretion of the clinical hepatology team based on the elevated inflammatory markers and lack of a standard of care for this phenotype. Therapy included intravenous methylprednisolone 10mg/kg/day for the first 3 days, transitioning to oral prednisone or prednisolone once discharged from the hospital, and tapering corticosteroids to off over 4 to 6 weeks. All clinical and research immune marker labs and liver biopsy samples were taken prior to the initiation of corticosteroids. Liver enzymes returned to normal for all patients by a median of 110 days (IQR 92–130) after hospital admission for activated T-cell hepatitis patients and by a median of 52 days (IQR 40–72) for indeterminate patients. At last follow up visit, a median of 330 days (IQR 275–559) for activated T-cell hepatitis patients and a median of 466 days (IQR 191–690) for indeterminate patients, all children were doing well with normal liver enzymes and no evidence of liver disease. No patients were receiving immunosuppressive medications.

## Discussion

We describe the first prospective study of peripheral blood T-cell phenotype in PALF patients with unknown etiology and identify significantly increased percentage of T_EM_ CD8+ CD103+ T-cells as a novel biomarker for patients with activated T-cell hepatitis. These results are consistent with our prior studies examining CD8 T-cells within both archived and fresh liver tissue samples from indeterminate PALF patients and suggest liver tissue and peripheral blood T-cell phenotypes may be similar in activated T-cell hepatitis [6, 7]. In the future, clinically available naïve and memory T-cell panels may be utilized to identify an increased percentage of CD8+ effector memory T-cells as a marker of patients with activated T-cell hepatitis. Similarly, lack of this phenotype should encourage consideration for an alternative diagnosis. The standard clinical T- and B-cell flow cytometry panel did not identify significant differences between groups, however there was a trend toward more activated T-cells (HLA-DR+) and a decreased CD4:CD8 T-cell ratio in the activated T-cell hepatitis patients. Lack of significance may be due to our small sample size, and these differences deserve further study. The use of surface CD45RA, CD103, and HLA-DR as markers to quantify CD103+DR+CD8+ T_EM_ cells may be a simpler, more widely available flow cytometry staining panel for differentiating PALF etiology than intracellular staining markers such a perforin that are more technically difficult to obtain. Our study limited the flow cytometry analysis to common markers of CD8 T-cell activation and phenotype of tissue residence. Multiplex staining helping to define a lymphocyte population phenotypically in conjunction with single cell transcriptional analysis is a rapidly developing field [11]. Using this technology for deep phenotyping of this population and its role in pathogenesis will be a focus of future work by our group.

Studies to date have shown the liver is a site of enrichment for CD103+ tissue resident memory T-cells where they play an important role in pathogen surveillance [12]. CD103 is an integrin that facilitates retention of the T-cells within tissues by binding to E-cadherin on epithelial cells. They are predominantly located in peripheral tissues, however upon stimulation they can recirculate into the blood where they may carry out systemic effector functions and attract immune cells to the liver [12]. In a recent adult study, CD8+ CD103+ T-cells were significantly increased in the liver of patients with autoimmune hepatitis, correlated with disease activity, and decreased after corticosteroid treatment [13]. Based on our study results, we are not able to determine whether the CD8+ CD103+ T-cells we identified are tissue resident memory T-cells circulating in the peripheral blood, or if CD103 is serving as a marker of another T-cell phenotype. Thus, the precise role of CD8+ CD103+ T-cells in PALF disease pathogenesis remains unknown and an area for future research.

Our results agree with findings from the Leonis et al. PALF Study Group study aimed to identify immune activation markers in PALF patients that were associated with outcomes. In that study, the combination of peripheral blood high perforin and granzyme expression in CD8 T-cells, increased absolute CD8 T-cell count, and increased sIL-2R level defined a unique high immune activation phenotype of patients who were more likely to have indeterminate diagnosis and decreased 21-day survival with native liver [14]. Notably, in our study despite considering an elevated sIL2R level as part of the criteria for activated T-cell hepatitis, we did not find a significant difference in median sIL2R level between groups. This may be related to small sample size (there was a numerical trend toward higher levels in the activated T-cell hepatitis patients) or may reflect sIL2R is a non-specific marker of T-cell activation that may be elevated in a number of different pathologic conditions. We now recognize T-cell HLA-DR positivity appears to be a more specific marker of T-cell activation to aid in differentiating between activated T-cell PALF and other etiologies to be used in the future. In addition, it is important to consider that other diagnoses may present with a phenotype of PALF with immune dysregulation and share similar features with activated T-cell hepatitis. These include viral hepatitis with prominent CD8 T-cell infiltration, drug-induced liver injury with hypersensitivity, autoimmune hepatitis, and hemophagocytic lymphohistiocytosis, and underscores the importance of a thorough diagnostic evaluation. In addition, genetic causes of immune dysregulation, including immunodeficiencies, need to be considered as part of the diagnostic workup and are increasingly being recognized as a cause of PALF especially among young children. Broad genetic testing was not performed for patients in our cohort, and thus we cannot exclude the possibility of an unidentified genetic diagnosis.

Peripheral blood flow cytometry results are a potential diagnostic tool that may be used in the future to identify PALF patients more or less likely to benefit from treatment with immunosuppressive therapy. Currently the benefit of immune-modulating treatment for activated T-cell hepatitis PALF patients remains unproven [4, 9]. In our study, all patients recovered with their native liver and it is unclear to what degree treatment with corticosteroids for the activated T-cell hepatitis group influenced this outcome. This question is the focus of the NIH funded multicenter, three-arm, randomized controlled Treatment for Immune Mediated Pathophysiology (TRIUMPH) trial which aims to determine whether suppressing inflammatory responses with either corticosteroids or anti-thymocyte globulin therapy improves survival for children with PALF of unknown etiology (U01DK062436). Results from our current study, among others, will inform mechanistic studies within the TRIUMPH trial, including the aim to better characterize peripheral blood and liver infiltrating T-cells in enrolled patients.

Finally, our study supports there are additional sub-phenotypes within the activated T-cell hepatitis group. One example is the subset of young children with PALF who often present with an immune-active phenotype and may be more likely to develop aplastic anemia [7, 14, 15]. The 3 young children in our study all had similar flow cytometry profiles with high percentages of CD8+ CD103+ T_EM_ cells that were HLA-DR+, had low CD4:CD8 T-cell ratios, and 2 of them developed aplastic anemia. A low CD4:CD8 T-cell ratio has been previously described in patients with hepatitis-associated aplastic anemia and may be a marker of increased risk of developing this complication [4, 16–18]. In contrast, the teenage patient with activated T-cell hepatitis had research flow cytometry results that were less skewed towards CD8+ CD103+ T_EM_ cells. Within the activated T-cell hepatitis group there are likely subsets of patients with different immune phenotypes, which may be related to the age of child when they experience the liver injury. How the developmental stage or relative immaturity of a child’s immune system influences the inflammatory responses need to be considered and requires further study with a broader age-range of patients. In addition, it has not yet been determined whether activated T-cell hepatitis is a disease entity in itself or a common final pathway for several hepatic insults, and perhaps young children are more susceptible to triggers that incite this type of liver-targeted immune activation. Further studies are needed to explore these age-specific and other differences within the PALF group.

Limitations of this study include the small number of patients enrolled and investigators were not blinded to diagnosis or immune-phenotype results. Classification of patients as activated T-cell hepatitis and treatment with corticosteroids was subjective and at the discretion of the treating clinicians. We identified a potential immune signature for the activated T-cell hepatitis group but given the small number of patients included this requires validation with larger cohorts. Furthermore, liver biopsy with CD8 staining, which was used as a criterion to diagnose activated T-cell hepatitis, was not available on all enrolled patients. It is possible that the two indeterminate patients who did not have a liver biopsy performed might have had prominent CD8 T-cell staining. Thus, we recognize that these groups were somewhat arbitrary and larger prospective studies are needed to support these preliminary findings. This is an evolving area of research and there is much still to learn about the activated T-cell hepatitis group. As hepatic CD8 T-cell infiltration and elevated sIL-2R levels can be seen in many different disease processes, we recognize that this is not an ideal way to define these patients. We hypothesize that in the future detailed flow cytometry results, such as those described here, among other to-be-determined characteristics, may better classify the phenotype of patients with activated T-cell hepatitis. Finally, we had hoped to compare individual patient fresh liver tissue and peripheral blood flow cytometry results, however, none of the patients in our study underwent liver transplantation, and this remains an area for future research.

## Conclusion

It is now well-established that an immune-active phenotype of indeterminate PALF exists, often referred to as immune-dysregulation and which we have classified as activated T-cell hepatitis. Our study identified a significant increase in peripheral blood CD8+ CD103+ effector memory T-cells as a novel biomarker for this group. In addition, our results suggest other widely available peripheral blood markers of CD8 T-cell activation and effector function, such as HLA-DR+, may be used to support this diagnosis and guide further studies. These results will inform ongoing translational research including the TRIUMPH trial. Future studies will aim to determine how flow cytometry can help identify subsets of PALF patients that could respond to different therapies and advance our understanding of the pathophysiology of this diverse disease process.

## Supporting information

S1 File (PDF)
