# Supplementary material for: Effector memory CD8 T-cells as a novel peripheral blood biomarker for activated T-cell pediatric acute liver failure
Source: PLoS One. 2023 Jun 2;18(6):e0286394. doi: 10.1371/journal.pone.0286394 (PMC10237500; doi:10.1371/journal.pone.0286394)
Supplement: S1 File — (PDF) [file pone.0286394.s001.pdf]

S1 Table. Demographic, laboratory, and clinical characteristics for all 10 enrolled patients

	All Patients n=10	Activated T-cell hepatitis n=4	Indeterminate PALF n=6	P value
At Study Enrollment				
Age, years	10 (4-14)	4 (3-7)	13 (9-15)	0.23
Gender, male	4 (40)	2 (50)	2 (33)	>0.99
Symptoms prior to admission, weeks	1.5 (0.5-2.8)	3.0 (2.8-3.0)	0.5 (0.5-0.9)	0.01
Hepatic encephalopathy level 0-1	6 (60)	3 (75)	3 (50)	0.57
Alanine aminotransferase (IU/L)	1715 (1396-3710)	1576 (1276-1965)	2879 (1477-7772)	0.35
Total bilirubin (mg/dL)	14.3 (4.1-17.5)	18.6 (17.5-19.8)	4.2 (1.9-11.6)	0.01
International normalized ratio	2.1 (2.0-2.2)	2.1 (2.0-2.1)	2.1 (2.0-2.3)	0.92
Total white blood cell count (1000/mm^3)	6.1 (3.5-9.0)	3.0 (2.2-4.9)	8.2 (5.3-9.0)	0.17
Hemoglobin (g/dL)	10.9 (10.1-12.1)	10.6 (10.2-11.6)	11.2 (9.6-12.1)	0.99
Platelet count (1000/mm^3)	183 (157-247)	183 (150-218)	199 (157-289)	0.65
Worst values during admission				
Hepatic encephalopathy 2-4	5 (50)	2 (50)	3 (50)	>0.99
Highest alanine aminotransferase (IU/L)	2381 (1643-6366)	1876 (1513-2249)	6068 (2619-8416)	0.26
Highest total bilirubin (mg/dL)	17.7 (5.7-21.6)	21.6 (20.4-22.6)	6.4 (2.2-13.3)	0.04
Highest international normalized ratio	2.3 (2.1-2.6)	2.3 (2.2-2.4)	2.3 (2.1-3.8)	0.88
Lowest total white blood cell count (1000/mm^3)	2.2 (1.4-7.3)	1.7 (1.3-5.5)	4.0 (1.7-7.3)	0.76
Lowest hemoglobin (g/dL)	10.1 (8.0-10.9)	10.3 (9.4-11.0)	9.7 (7.8-10.8)	0.76
Lowest platelet count (1000/mm^3)	133 (57-223)	140 (100-179)	105 (57-229)	>0.99
Outcome and follow up				
Duration of admission, days	11.5 (7.3-14.8)	9.0 (7.8-11.3)	13.5 (6.3-18.5)	0.76
Admission to normal liver enzymes, days	81.0 (46.5-92.0)	110.0 (92.0-130.0)	52.0 (36.5-84.0)	0.11
Immune studies				
Soluble IL-2 receptor (IU/mL)	1916 (1311-5112	4588 (1689-8653)	1664 (995-2132)	0.26
Perforin and Granzyme B Assay, n	8	4	4	
% CD8 T-cells Perforin+	18.5 (8.0-27.0)	29.5 (18.5-44.0)	11.5 (7.0-16.5)	0.34
% CD8 T-cells Granzyme+	36.5 (16.8-57.8)	60.5 (44.5-70.0)	25.0 (14.3-34.3)	0.20
Clinical T & B cell quantitation				
CD4 to CD8 T-cell ratio	1.3 (0.5-1.7)	0.3 (0.3-0.7)	1.5 (1.2-1.9)	0.11
% CD8+ T-cells	31.0 (25.5-33.8)	34.5 (26.5-40.3)	30.0 (25.5-33.0)	0.45
% HLA-DR+ T-cells	12.0 (5.0-27.3)	32.0 (23.8-37.3)	8.0 (5.0-12.5)	0.11
Research Flow cytometry				
CD4 to CD8 T-cell ratio	1.5 (1.2-1.8)	0.8 (0.3-1.6)	1.6 (1.5-1.8)	0.26
% CD8+ T-cells	36.4 (31.7-42.0)	46.3 (34.7-56.2)	34.5 (31.7-37.1)	0.26
% Effector memory CD8+ T-cells	29.5 (16.7-57.4)	66.8 (57.4-68.7)	19.1 (14.1-27.5)	0.02
% Effector memory CD8+ CD103+ T-cells	8.8 (5.3-15.3)	12.4 (9.5-14.7)	5.8 (4.7-15.0)	0.48

[a]Data are medians (interquartile range) or n (%).

S2 Table. Clinical and research immune studies data for 3 younger activated T-cell hepatitis patients compared to indeterminate PALF patients

	**Activated T-cell hepatitis ≤ 5y n=3**	**Indeterminate PALF n=4**	**P value**
Soluble IL-2 receptor (IU/mL)	7430 (4588-9875)	2116 (1875-3135)	0.4
Perforin and Granzyme B Assay, n	3	2	
% CD8 T-cells Perforin+	36.0 (29.5-52.0)	16.5 (12.8-20.3)	0.4
% CD8 T-cells Granzyme+	66.0 (60.5-74.0)	25.0 (21.5-28.5)	0.2
Clinical T & B cell quantitation			
CD4 to CD8 T-cell ratio	0.3 (0.2-0.3)	1.8 (1.5-2.6)	0.06
% CD8+ T-cells	40.0 (34.5-40.5)	26.0 (21.3-28.5)	0.11
% HLA-DR+ T-cells	34.0 (32.0-40.5)	8.0 (4.8-11.5)	0.06
Research Flow cytometry			
CD4 to CD8 T-cell ratio	0.4 (0.3-0.8)	1.8 (1.7-3.3)	0.06
% CD8+ T-cells	54.4 (46.3-58.0)	32.4 (26.1-34.1)	0.06
% Effector memory CD8+ T-cells	67.2 (66.8-70.2)	19.1 (13.4-25.2)	0.06
% Effector memory CD8+ CD103+ T-cells	14.4 (12.4-15.0)	4.7 (4.5-5.3)	0.06

[a]Data are medians (interquartile range).
